# The Value of Contrast-Enhanced Ultrasound-Guided Contrast Injection via the Endoscopic Nasobiliary Drainage Duct in Diagnosing Residual Common Bile Duct Stones

**DOI:** 10.1155/2020/3281241

**Published:** 2020-07-01

**Authors:** Yang Wang, Yue Yang, Kaiming Wang, Shaoshan Tang

**Affiliations:** ^1^Department of Ultrasound, Shengjing Hospital of China Medical University, Shenyang, Liaoning, China; ^2^Department of Orthopedic Surgery, Shengjing Hospital of China Medical University, Shenyang, Liaoning, China

## Abstract

**Objectives:**

To investigate the diagnostic value of contrast-enhanced ultrasound- (CEUS-) guided contrast injection via an endoscopic nasobiliary drainage (ENBD) tube on the evaluation for residual stones in common bile duct (CBD).

**Methods:**

116 patients with CBD stones were treated by endoscopic retrograde cholangiopancreatography and duodenoscopic sphincterotomy incision surgery and ENBD. The US group consisted of 54 patients who underwent US-guided saline injection via the ENBD tube to evaluate for residual CBD stones. The CEUS group consisted of 62 patients who underwent CEUS-guided contrast injection via the ENBD tube to evaluate for residual CBD stones. The length and width of CBD and the detection rate of residual stones before and after NS injection were compared.

**Results:**

In both the US group and the CEUS group, the rate of complete demonstration and the average length and width of CBD before and after injection of NS were all increased significantly. In the US group, 6 patients had verified residual stones, 1 of which was detected by conventional US (detection rate, 1/6), 5 of which were detected by saline injection ultrasound (detection rate, 5/6), and 4 of which were detected by ENBD cholangiography (detection rate, 4/6). There was 1 false positive result on saline injection US and 2 false positives on ENBD cholangiography. In the CEUS group, 6 patients had verified residual stones, none of which were detected by conventional US (detection rate, 0/6), all of which were detected by saline injection CEUS (detection rate, 6/6), and 4 of which were detected by ENBD cholangiography (detection rate, 4/6). There was 1 false positive result on ENBD cholangiography.

**Conclusions:**

CEUS-guided contrast injection via an ENBD tube helps to provide clear observation of residual stones in the CBD after ERCP with EST and provides comprehensive information for follow-up.

## 1. Introduction

Gallstones are common and affect 10–15% of the adult population. Some 4% of these patients become symptomatic each year, with biliary colics, cholecystitis, or cholangitis [1]. There are currently 2 widely accepted treatment strategies for patients presenting to the hospital with choledocholithiasis, including endoscopic retrograde cholangiopancreatography (ERCP) and laparoscopic exploration of the common bile duct (CBD) [2]. Historically, ERCP provided a less invasive method for treating choledocholithiasis. With improvements in endoscopic technology and instruments, ERCP with endoscopic sphincterotomy (EST) has gradually become the mainstream method of treatment [3].

Cotton et al. [4] were the first to use an endoscopic nasobiliary drainage (ENBD) catheter for several days in the common bile duct (CBD) after endoscopic sphincterotomy (EST). ENBD placement provides reliable biliary drainage and perfusion and allows for cholangiography. ENBD also reduces the need for instrumental stone extraction and repeated endoscopy and transnasal cholangiography to assess whether the stones have been fully cleared [5]. In subsequent studies [6–10], it was found that EST or endoscopic papillary balloon dilatation (EPBD) followed by ENBD reduces the incidence of postendoscopic retrograde cholangiopancreatography (ERCP) complications, such as pancreatitis and cholangitis, particularly in patients with persistent stones, infected bile, or blood clots in the biliary tree. Nasobiliary cholangiography is often performed after stone extraction to confirm complete clearance of the bile duct, but the radiation exposure involved with X-ray and the possibility of an anaphylactic reaction to iodinated contrast media restricts its application. Routine ultrasound (US) has many advantages for diagnosing issues with the bile duct system, but it is not ideal for evaluating the CBD after ERCP because of abdominal gas and surgical trauma. Thus, we describe herein a new method for contrast-enhanced ultrasound- (CEUS-) guided contrast injection via an ENBD tube to evaluate for residual CBD stones and explore its value for clinical application.

## 2. Materials and Methods

### 2.1. Patients

The records of 116 patients with CBD stones who underwent ERCP and EST with ENBD tube placement were retrospectively reviewed. All patients gave written informed consent for study inclusion. The US group consisted of 54 patients who underwent US-guided saline injection via the ENBD tube to evaluate for residual CBD stones between April 2015 and January 2016. The mean age in the US group was 59.3 ± 16.7 years (range, 21–83 years), and there were 24 men and 30 women. The CEUS group consisted of 62 patients who underwent CEUS-guided contrast injection via the ENBD tube to evaluate for residual CBD stones between February 2016 and December 2016. The mean age in the CEUS group was 55.9 ± 14.4 years (range, 25–88 years), and there were 31 men and 31 women.

### 2.2. Equipment and Agent

The ultrasound device (Aplio 500; Toshiba Corporation, Japan) was used with a 3 to 5 MHz transducer. The contrast agent (SonoVue; Bracco S.p.A., Milan, Italy) was administered as a 2.4 mL bolus followed by a 5 mL saline flush. After mixing, the agent was diluted to 1 : 100 for injection. All patients refrained from food and water intake for 6 hours before evaluation, and all ultrasound examinations were carried out by a physician with more than 20 years of experience, 1 to 2 days after ERCP.

All ERCP and EST procedures were performed by experienced therapeutic endoscopists (>300 cases/year and >15 years of experience each). Iodine contrast examination was conducted after stone extraction to detect any residual stones. If there was a significant filling defect, the stone extraction would be repeated until no residual stones were detected. An ENBD tube was placed once no significant filling defect was detected.

Routine postoperative US examinations were performed to detect any residual stones in the CBD and to record the length and width of the CBD. In the US group, saline was injected via the ENBD tube into the CBD for the dynamic observation of suspected residual stones. If dilation of the CBD was required, large boluses of saline were injected over a short time. The size and location of stones, the length and width of the CBD, and the amount of injected saline were recorded. In the CEUS group, the imaging mode was switched to low mechanical index contrast-specific imaging, and the contrast agent was injected slowly. The location and shape of the CBD were verified by tracing the imaging results. Using CEUS imaging guidance and with the transducer maintained in a stable position, saline was injected via the ENBD tube into the CBD. The size and location of stones, the length and width of the CBD, and the amount of contrast agent and saline were recorded. Nasobiliary cholangiography was performed within 1 day after the ultrasound procedure.

All patients with residual stones suspected by the results of any of the above examinations underwent ERCP or open surgery to verify the existence of residual stones after 3 to 5 days.

All patients underwent follow-up within 3 days after the examination to evaluate for pain, fever, allergic reaction, or other adverse effects. Patients were monitored at 3 to 5 months after the procedure for residual stones.

### 2.3. Evaluation Criteria

The presence of residual stones was defined as a hyperechoic mass in the CBD, without deformation, detected during flushing. Patency of the CBD was determined when contrast agent flowed smoothly into the duodenum, and there was no resistance during injection. The degree of demonstration of the CBD was categorized as follows: (1) unclear—the location and shape could not be confirmed after repeated scanning; (2) partial demonstration—US could only demonstrate a part of the CBD; and (3) complete demonstration—US could demonstrate the entire CBD from the superior duodenal segment to the pancreatic segment.

### 2.4. Statistical Analysis

Data were analyzed using SPSS software (SPSS Inc., Chicago, IL, USA). Quantitative data were expressed as the mean ± standard deviation. A paired *t*-test was used to compare the change in the width and length of the CBD before and after saline injection. *P* values < 0.01 were considered statistically significant.

## 3. Results

### 3.1. Dosage of Saline and Contrast Agent

The dose of saline was 133.3 ± 76.74 mL in the US group and 57.22 ± 33.51 mL in the CEUS group. The dose of contrast agent in the CEUS group was 14.91 ± 12.19 mL.

### 3.2. CBD Demonstration

Of the 54 patients in the US group, 18.5% (10/54) had complete demonstration of the CBD by routine ultrasound. After injection of water into the CBD via the ENBD tube, the rate of complete demonstration increased to 87.0% (47/54) (Table 1).

Of the 62 patients in the CEUS group, contrast injection failed in 1; this was later determined to be an obstruction of the ENBD tube. Of the 61 patients who underwent successful injection, the rate of complete demonstration on routine US was 13.1% (8/61). After injection into the CBD via the ENBD tube under CEUS guidance, the rate of complete demonstration increased to 86.9% (53/61) (Table 2).

### 3.3. Observed Length and Width of the CBD

The average observed length and width of the CBD increased significantly after saline injection in both the US group and the CEUS group (Table 3).

### 3.4. Detection of Residual Stones

**Figure 1:**
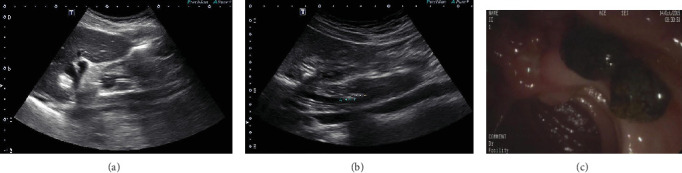
(a) US could only demonstrate the upper part of the CBD before saline injection. (b) The CBD were completely demonstrated after saline injection and two hyperechoic masses were found at the end of the CBD. (c) Two tawny stones were detected by ERCP.

In the US group, 45 patients had no residual stones detected by conventional US, saline injection US, or ENBD cholangiography, and there were no recurrences after 3 to 5 months of follow-up. The 9 patients who had suspected residual stones underwent ERCP. Six patients had verified residual stones, 1 of which was detected by conventional US (detection rate, 1/6), 5 of which were detected by saline injection ultrasound (detection rate, 5/6), and 4 of which were detected by ENBD cholangiography (detection rate, 4/6). There was 1 false positive result on saline injection US and 2 false positives on ENBD cholangiography (Figure 1).

**Figure 2:**
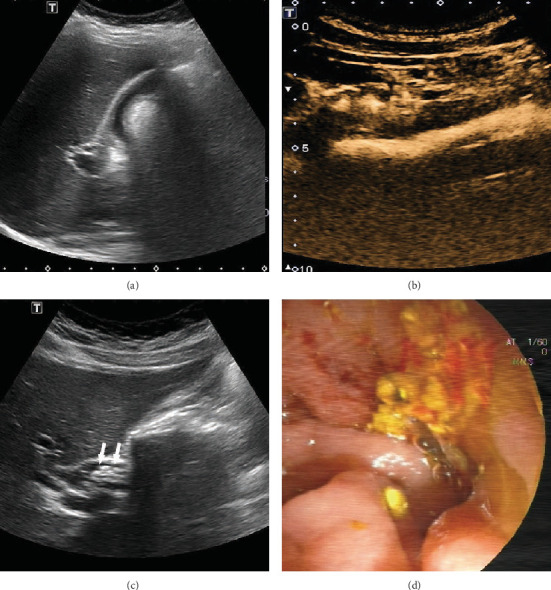
(a) US could not demonstrate the CBD clearly before saline injection. (b) The morphology, position, and course of the CBD were displayed by CEUS imaging. (c) The CBD were completely demonstrated after injection, and several hyperechoic masses were found in the beginning of the CBD. (d) Several tawny stones were detected by ERCP.

In the CEUS group, 55 patients had no residual stones detected by conventional US, contrast-injection CEUS, or ENBD cholangiography, and there were no recurrences after 3 to 5 months of follow-up. The 7 patients who had suspected residual stones above underwent ERCP. Six patients had verified residual stones, none of which were detected by conventional US (detection rate, 0/6), all of which were detected by saline injection CEUS (detection rate, 6/6), and 4 of which were detected by ENBD cholangiography (detection rate, 4/6). There was 1 false positive result on ENBD cholangiography (Figure 2).

### 3.5. An Incidental Finding

**Figure 3:**
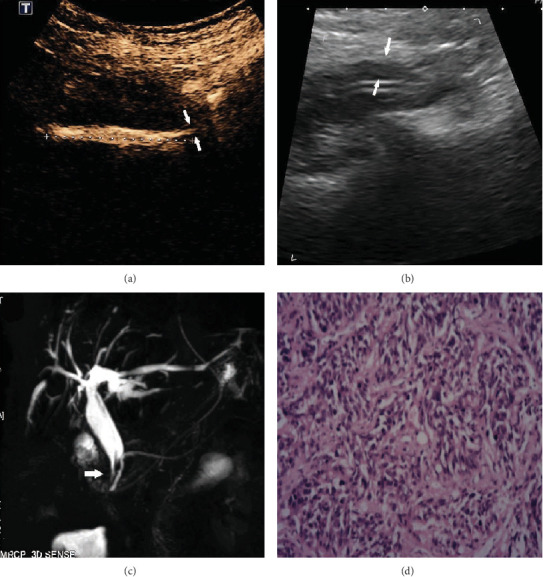
(a) The CEUS image became narrow at the end of the CBD consistent with the “bird's beak sign.” (b) Saline injection revealed that the wall at the end of the CBD was thickened. (c) ENBD cholangiography showed a filling defect (narrow). (d) The results of surgical pathology revealed adenocarcinoma.

In the CEUS group, a single patient experienced slow flow of the contrast agent into the duodenum, with narrowing at the end of the CBD consistent with the “bird's beak sign.” Saline injection revealed that the wall at the end of the CBD was thickened, and ENBD cholangiography showed a filling defect. The patient underwent magnetic resonance imaging and later magnetic resonance cholangiopancreatography, which indicated uneven thickening at the head of the pancreas. The results of surgical pathology revealed adenocarcinoma (Figure 3).

## 4. Discussion

Since EST at the time of ERCP was first described in 1974, its use has been advocated for elderly patients or those with comorbid illness excluding them from surgical management [11–13]. Endoscopic removal of CBD stones using ERCP with biliary sphincterotomy or papillary balloon dilation is more widely used than the open approach due to its higher success rate and lower morbidity [14]. However, the operational maneuvers of ERCP, especially EST and stone extraction, carry certain risks such as acute pancreatitis and biliary sepsis. Numerous studies have reported that ENBD following endoscopic treatment can reduce pressure within the bile duct, reducing endotoxin and bacterial accumulation in the blood and thereby improving patients' prognosis [7, 10, 15].

Several investigators have described recurrent stones as a complication after EST, with the frequency varying widely—from 4% to 24%—and the recurrence occurring as soon as 4 months after complete extraction of bile duct stones [16, 17]. Therefore, several endoscopists advocate for the desirability of preventing retained residual stones by confirming their absence using cholangiography via the ENBD tube [18–20]. However, when residual stones measure less than 4 mm or when the bile duct is dilated, cholangiography may miss residual stones [21, 22]. The clinical application of ENBD cholangiography is also limited by the associated radiation exposure and the potential for allergic or toxic reactions to the iodide contrast agent.

Conventional US, as the first-line imaging for biliary disease, can display the number and location of stones and can confirm expansion of the bile duct. However, due to the location of the CBD and its particular structure, US images are easily interfered with by abdominal fat, gastrointestinal gas, the omentum, and other sources of mixed echoes that reduce visualization, especially of the middle and lower segments of the CBD. Moreover, the CBD is relatively open after ERCP because of the surgical trauma, making the interference from gastrointestinal gas more obvious, resulting in a further decrease in CBD visibility and an increase in the difficulty of inspection.

In our US group, the length, width, and rate of complete demonstration of the CBD all increased significantly after saline injection through the ENBD, expanding the diagnostic horizon and allowing for detection of small stones that might be missed by conventional US. Taking advantage of the saline flow, the number and location of residual stones in the CBD can be observed dynamically. Gas in the CBD can be pushed away by the flow of saline, combined with the change in body position. This is conducive to determining the physical characteristics of any lesions and allows identification of an echo between the air mass and any stones. In our US group, all but 1 of 14 patients that could not be diagnosed due to the unclear appearance of the CBD on conventional US were diagnosed after saline injection. The detection rate of saline injection US is significantly higher than conventional US and slightly higher than ENBD cholangiography. However, for patients with more intestinal gas and other interfering factors, it is necessary to infuse a large amount of saline over a short time. This expands the CBD rapidly, allowing for clarification of its course, but this procedure must often be repeated. Injecting a large volume of fluid causes pain in patients after ERCP, from both the nasopharyngeal foreign body and the expansion of the biliary tract; there is also a risk of complications.

Ultrasound contrast agents are essential in transabdominal ultrasonography for the investigation of several abnormalities, in particular for the differential diagnosis of solid liver lesions and also widely widespread in EUS to enhance its diagnostic accuracy, mainly in pancreaticobiliary diseases [23]. Intracavitary CEUS, in which the contrast agent is administered through a drainage tube or natural orifice, is becoming more commonly mentioned in recent years [24–26]. The CEUS imaging through an ENBD can clearly display the morphology, position, and course of the CBD, and the imaging results play a role in guidance and positioning for saline injection. Of the 61 patients in our CEUS group with successful injection, the CEUS imaging of the CBD demonstrated its position and course. This guided injection greatly improves the complete demonstration rate of the CBD and reduces the amount of saline injected, relieving pain and reducing the risk of complications. Our CEUS group required a reduced dose of saline relative to our US group. In addition, only 2 of the 11 patients that could not be diagnosed due to an unclear CBD appearance on conventional US remained undiagnosed after saline injection guided by CEUS. The detection rate of saline injection US is significantly higher than that of conventional US and slightly higher than that of cholangiography.

It is worth mentioning that CEUS can determine the patency of the CBD by showing whether and how quickly the contrast agent is flowing into the intestine. One patient in our CEUS group was not able to be imaged after the injection of the contrast agent; this proved to be because of a blockage of the drainage tube. In another patient, the contrast agent entered the intestine slowly, and the end of the CBD appeared as a “bird's beak.” This was proven to be because of adenocarcinoma after surgery and pathologic examination.

The reason for the single false positive in our US group may be related to postoperative cholestasis and tissue deposition adherent to the bile duct. The 3 false positives on cholangiography in both the US group and the CEUS group may be related to bubble shadow.

Despite the usefulness of this method, we were left with 1 patient with unclear demonstration and 6 patients with partial demonstration in the US group and 2 patients with unclear demonstration and 6 patients with partial demonstration in the CEUS group. The reasons for this may be as follows: (1) severe interference caused by gastrointestinal gas related to the age of the patient; (2) the relatively open state and structural changes of the biliary system after laparoscopic cholecystectomy, leading to poor-quality US images; (3) excessive incision of the duodenal papillary sphincter during surgery, causing immediate flow of both contrast agent and saline into the intestinal cavity and not allowing for CBD expansion; and (4) inflammation of the biliary system creating a less elastic bile duct wall that cannot fully expand.

The limitations of our study include the fact that we did not track patients who were not suspected of harboring residual stones on conventional US, saline injection US, or ENBD cholangiography. We need to improve our long-term follow-up to track whether any residual stones are, in fact, present in these patients. Besides, the interval between US/CEUS of the CBD, subsequent cholangiography, and ERCP may have contributed to difference in the detection and number of stones which can be explained by the time-dependent stone redistribution (i.e., spontaneous migration into the duodenum or further passage of stones from the gallbladder) [27–29]. The shortest possible interval is required in a further study.

In conclusion, CEUS-guided contrast injection via an ENBD tube helps to provide clear observation of residual stones in the CBD after ERCP with EST and provides comprehensive information for follow-up. This method provides economic benefit, is convenient and noninvasive, and has no associated radiation exposure: a series of advantages with high clinical significance and value for postoperative patients with an ENBD tube.

## Figures and Tables

**Table 1:** Demonstration of the common bile duct in the US group.

	Routine ultrasound	Saline injection
Unclear	14 (25.9%)	1 (1.9%)
Partial demonstration	30 (55.6%)	6 (11.1%)
Complete demonstration	10 (18.5%)	47 (87.0%)

**Table 2:** Demonstration of the common bile duct in the CEUS group.

	Routine ultrasound	Contrast injection
Unclear	11 (18.0%)	2 (3.3%)
Partial demonstration	42 (68.9%)	6 (9.8%)
Complete demonstration	8 (13.1%)	53 (86.9%)

**Table 3:** Average observed length and width of the common bile duct before and after saline injection.

	US group		CEUS group	
	Length (cm)	Width (cm)	Length (cm)	Width (cm)
Before injection	2.63 ± 0.26	0.49 ± 0.35	2.94 ± 1.76	0.58 ± 0.30
After injection	5.79 ± 2.17	0.99 ± 0.35	6.09 ± 1.46	1.11 ± 0.98
*t* value	11.03	10.81	12.39	3.91
*P* value	<0.01	<0.01	<0.01	<0.01

## Data Availability

All data included in this study are available upon request by contact with the corresponding author.
